# Modeling and Optimization of Connected and Automated Vehicle Platooning Cooperative Control with Measurement Errors

**DOI:** 10.3390/s23219006

**Published:** 2023-11-06

**Authors:** Weiming Luo, Xu Li, Jinchao Hu, Weiming Hu

**Affiliations:** 1 School of Instrument Science and Engineering, Southeast University, Nanjing 210096, China; 230188092@seu.edu.cn (W.L.); 230189688@seu.edu.cn (J.H.);; 2 China Automotive Engineering Research Institute Company Ltd., Chongqing 401122, China

**Keywords:** intelligent transportation, connected and automated vehicle (CAV) platoon, sensor measurement error, cooperative control

## Abstract

This paper presents a cooperative control method for connected and automated vehicle (CAV) platooning, thus specifically addressing the challenge of sensor measurement errors that can disrupt the stability of the CAV platoon. Initially, the state-space equation of the CAV platooning system was formulated, thereby taking into account the measurement error of onboard sensors. The superposition effect of the sensor measurement errors was statistically analyzed, thereby elucidating its impact on cooperative control in CAV platooning. Subsequently, the application of a Kalman filter was proposed as a means to mitigate the adverse effects of measurement errors. Additionally, the CAV formation control problem was transformed into an optimal control decision problem by introducing an optimal control decision strategy that does not impose pure state variable inequality constraints. The proposed method was evaluated through simulation experiments utilizing real vehicle trajectory data from the Next Generation Simulation (NGSIM). The results demonstrate that the method presented in this study effectively mitigates the influence of measurement errors, thereby enabling coordinated vehicle-following behavior, achieving smooth acceleration and deceleration throughout the platoon, and eliminating traffic oscillations. Overall, the proposed method ensures the stability and comfort of the CAV platooning formation.

## 1. Introduction

A connected and automated vehicle (CAV), also known as an intelligent connected Vehicle, represents an emerging product within the realm of vehicular networking within the transportation sector. The CAV is set to facilitate the emergence of new forms and modes of transportation operations. The platooning mode of CAVs represents one of the archetypal applications in this domain. In the CAV platoon system, advanced onboard sensors and communication technologies are deployed for the purpose of detecting and exchanging information concerning the operational status of the vehicles. Through the application of CAV platoon control techniques, these CAVs are orchestrated to maintain a close spatial arrangement, thereby operating in a platoon formation. This operational configuration holds the potential to enhance road capacity, reduce energy consumption, and carry various latent advantages [1].

Traditional vehicle platoon control primarily relies on adaptive cruise control (ACC) technology. Building upon the foundation of cruise control (CC), ACC technology utilizes onboard sensors such as millimeter-wave radar and cameras to gather real-time information about the preceding vehicle’s speed, acceleration, and headway distance. Then, this data is used to regulate the throttle and brake systems, thereby enabling speed control within a following scenario and maintaining a safe distance from the leading vehicle. However, platoon operation lacks a coordinated mechanism, which results in an inability to mitigate traffic shockwaves originating from upstream traffic. This is due to exclusive reliance on the motion states of the preceding and following vehicles for formulating control decisions in the absence of real-time status data from other vehicles within the platoon. This deficiency leads to a subpar performance in controlling the platoon system under complex operational conditions [2]. In response to the aforementioned issues, scholars have introduced the cooperative adaptive cruise control (CACC) technology, which encompasses cooperative-perception-based CACC methods and cooperative-behavior-based CACC methods [3]. Dhawankar et al. [4] have addressed the development and numerical implementation of a V2X (vehicle-to-vehicle and vehicle-to-infrastructure) control system architecture for an autonomous vehicle platoon. Their investigation encompassed a comprehensive set of case studies aimed at evaluating the system’s performance in various aspects, including its responsiveness to the communication infrastructure, sensitivity to emergency scenarios, adaptability under conditions of communication loss, and its behavior in dynamically changing driving environments. Lazar et al. [5] present a comprehensive overview of the control architecture for connected vehicle platoons, wherein they discuss sensor technologies, in-vehicle networks, vehicular communication, and control solutions with the goal of improving road safety, traffic flow, emissions, fuel consumption, and driver comfort. The cooperative-perception-based CACC methods primarily utilize the preceding vehicle’s speed information [6], information regarding the positions and speeds of multiple preceding vehicles [7], or the position information of both preceding and following vehicles [8] to optimize the control performance of individual vehicles within the platoon. However, these cooperative-perception-based CACC methods do not consider all the vehicles within the platoon as a cohesive unit; thus, they are effectively lacking in true collaboration, which affects the platoon stability [9]. On the other hand, the cooperative-behavior-based CACC methods treat all CAVs in the platoon as an integrated whole, thus achieving synchronized control of the longitudinal driving behavior (acceleration or deceleration) in the platoon system to maximize control performance [10]. Generally, cooperative-behavior-based CACC methods transform the platoon control problem into an optimal control problem for overall optimization. These methods utilize measurements of the current platoon state to predict future system dynamics and optimize performance metrics, thereby determining the optimal control decisions for all follower vehicles. By incorporating various optimization objectives and constraints, these methods systematically enhance comfort, safety, energy efficiency, and other performance aspects [11]. Ren et al. [12] proposed a CACC algorithm based on the frenet frame, which decouples the vehicle motion into one-dimensional motion in order to simplify the controller design and improve the efficiency of the solution. Tan et al. [13] proposed a real-time predictive distributed CACC control framework that addresses time delays, actuator lag, and utilizes intent-sharing-based distributed computing to improve string stability under various traffic dynamics by formulating a Kalman-filter-based real-time current driving state prediction model, thus solving the problem using a sequential Kalman filter update process and implementing a real-time distributed MPC-based CACC controller with delay-compensated predicted initial conditions. Existing research indicates that, when compared with noncooperative-behavior-based models, cooperative-behavior-based CACC methods can provide smoother acceleration and deceleration behaviors, thereby ultimately enhancing the stability and safety of CAV platoon systems [14].

In the literature, the design of CACC is based on the assumption that sensors can acquire high-precision motion state data for a CAV platoon. Thus, the sensor uncertainty remains a significant challenge for the deployment of CAV platoons in real-world road traffic environments [15]. Tian et al. [16] designed a novel controller based on an interval type-3 fuzzy logic system to address the challenges of the CAV’s lateral path tracking, which handles uncertainties and tackles approximation errors and perturbations using compensators. Additionally, the suggested adaptation laws estimate the bounds of uncertainty and ensure stability under unknown dynamics and critical maneuvers. Focusing on improving the lateral motion performance under different maneuvers and in the presence of parameter uncertainties and external disturbances, the article of [17] introduced a robust two-layer control scheme that enhanced the performance of four-wheel independent-drive electric vehicles. Moreover, in the logical architecture of a CAV platoon system, the acquisition of data from onboard sensors is a pivotal step, which relies on various onboard sensors to continuously measure and obtain the platoon’s motion state [18]. However, in practical driving scenarios, onboard sensors may experience failures or exhibit significant measurement errors over certain time intervals, thereby negatively affecting the control efficacy of CACC algorithms. This could potentially introduce uncertainty in future vehicle states, thereby leading to reduced platoon stability, oscillations, or even traffic accidents. Li et al. [19] proposed an improved modified model predictive control (MPC) method for energy-optimal ACC that addresses the negative effects of system noise. Andrade, E. et al. [20] introduced a PReCAV, which is a mechanism for cooperative CAV platoon recovery against false data injection attacks, thus utilizing virtual and physical system models to maintain platoon stability and resilience, as well as ensuring stability and resilience in the face of network and sensor attacks. Cui et al. [15] presented a simulation platform that enables the assessment of CACC impacts on safety, thus considering factors such as vehicle dynamics, sensor errors, automated vehicle control algorithms, crash severity, and cybersecurity attacks. The issue of sensor failures in CAV platoons is a current area of research focus [21]. For instance, Wang et al. [22] proposed a robust, nonfragile, fault-tolerant control strategy to ensure the functional safety of CACC in the case of sensor failures. Simulation experiments demonstrated the effectiveness of this quantitative risk reduction method for platoon driving. Cai et al. [23] addressed sensor failure in complex traffic scenarios, which effectively controls the speed of the CAV platoon and prevents collisions. Guo et al. [24], based on a switched sampling data CACC system model, proposed a state feedback controller design approach that ensures the stable stability of the system and mitigates the impact of sensor failures. Regarding onboard sensor measurement errors, Chen et al. [25] introduced a preceding vehicle recognition method within a fully networked vehicle environment for mitigating sensor measurement errors in real traffic conditions. However, experimental findings derived from the NGSIM real vehicle trajectory data underscored the necessity for ensuring a relative position accuracy within a 1.1 m threshold to ensure collision avoidance through the method. Yang et al. [26] proposed a robust H controller to mitigate the combined impacts of sensor fusion errors and sensor/channel noise and their effect on platooning performance, thus including the tracking performance and string stability. Zhou et al. [27] focused on optimizing the car-following stability of CAV platoons under periodic disturbance through an autonomous platoon formation strategy (APFS), which contributed to the understanding of CAV platooning strategies and highlighted the potential of APFS to improve the stability of CAV platoons in real-world traffic scenarios. Sheikh et al. [28] primarily investigated collision avoidance for the on-ramp merging of autonomous vehicles and proposed a collision avoidance model that effectively reduces collision risks and improves traffic safety. Additionally, some researchers employ techniques such as deep learning [29] and reinforcement learning [30] to enhance the performance of CAV platoons under control delays and sensor measurement errors. Nonetheless, these methods require large datasets for training and face generalization challenges.

**Figure 1:**
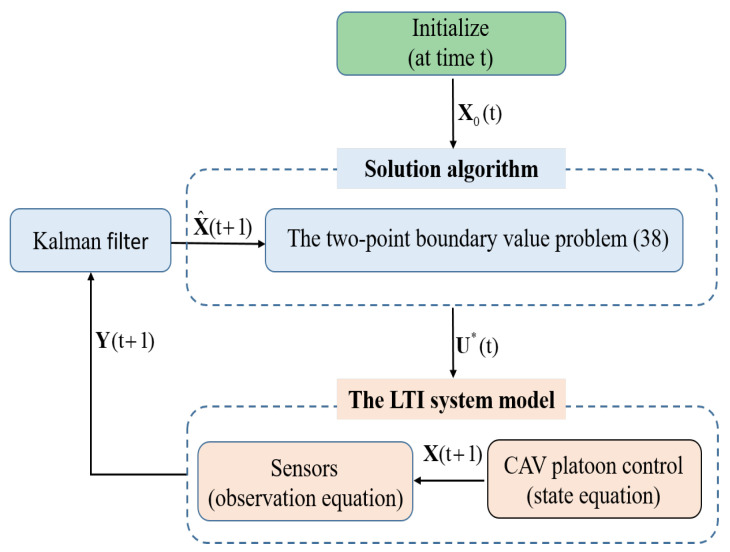
The conceptual flowchart of the proposed method.

Although the existing research on CACC has touched upon the issue of sensor measurement errors, there has been a lack of in-depth exploration into the impact mechanism of measurement errors on the collaborative control of CAV platoon systems. This gap renders CACC methods less adaptable to intricate road traffic operational environments. Addressing these concerns, this study establishes a cooperative-behavior-based CACC state-space system model that accounts for onboard sensor measurement errors, thereby utilizing a fixed time interval strategy that substantiates the cumulative effects of multiple sensor measurement errors. Moreover, by employing Kalman filtering to mitigate the negative impact of measurement errors, a strategy is proposed for optimal control decision making without the constraints of pure state variable inequalities. As shown in Figure 1, the initialization of the parameters, size, and state variables $X_{0}$(t) of the CAV platoon occurs at time t. The leading CAV obtains the values of $X_{0}$(t) through vehicle-to-vehicle (V2V) communications. The solution algorithm solves the two-point boundary-value problem (38) to determine the optimal control decisions $U^{*}$. The optimal control decisions are sent from the leading CAV to the following CAVs to control the CAV platoon. The state variables X(t+1) for the CAVs are observed by the onboard sensors. Kalman filtering is employed to estimate the value of $\hat{X}$(t+1) for the motion state of the CAV platoon. Then, the two-point boundary-value problem (38) is solved again to obtain the optimal control decisions at time t+1. These steps are repeated at each sampling time step. This strategy aims to ensure the smooth operation of the CAV platoon system, thus safeguarding both the platoon stability and comfort. The theoretical contributions of this article can be summarized as follows:

(1) Introduction to the cooperative control method considering sensor measurement errors: The article proposes a cooperative control method that takes into account sensor measurement errors in the context of CAV platooning. By analyzing the cumulative effects of sensor measurement errors and introducing the application of Kalman filtering, the proposed method effectively mitigates the adverse impacts of measurement errors and enhances the stability and control performance in CAV platooning.

(2) Transformation from the CAV platooning formation control problem into an optimal control decision problem: The article transforms the CAV platooning formation control problem into an optimal control decision problem by introducing an optimal control decision strategy that does not rely solely upon pure-state variable inequality constraints. This approach enables the optimization of control decisions for the platoon while ensuring smooth acceleration and deceleration, thereby eliminating traffic oscillations and maintaining stability and comfort.

(3) Validation of the proposed methodology through simulation experiments: The article evaluates how effective the proposed cooperative control method is through simulation experiments that utilize real vehicle trajectory data, which represents the movement of the leading CAV. The results demonstrate how the proposed method successfully mitigates the influence of measurement errors and enables coordinated vehicle-following behavior, achieves smooth acceleration and deceleration throughout the platoon, and eliminates traffic oscillations. Overall, the proposed method ensures the stability and comfort of the CAV platooning formation.

## 2. Cooperative-Behavior-Based CACC State-Space Model with Measurement Errors

### 2.1. Assumptions

**Figure 2:**
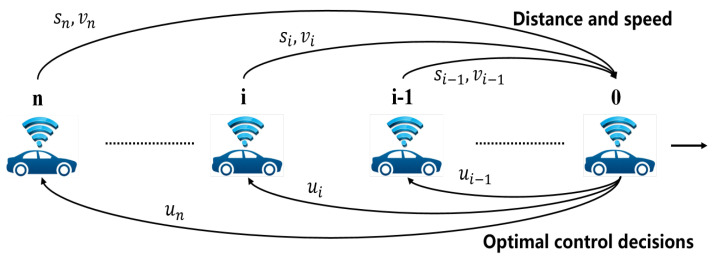
The platoon of CAVs.

Consider the CAV platoon system in a highway lane as illustrated in Figure 2, where $i\in \left\{0,1,2,\cdots ,n\right\}$ represents the longitudinal serial number of the CAVs in the platoon stream ranging from 0 for the leading vehicle to n for the tail vehicle. The following assumptions will be employed to design the longitudinal control of the CAV platoon system:

(1) All vehicles in the platoon stream are CAVs.

(2) All CAVs are equipped with onboard sensors that are capable of collecting motion-state data, such as speed and relative distance to the preceding vehicle. The models of onboard sensors are the same in the CAV platoon.

(3) Bidirectional vehicle-to-vehicle (V2V) communication is established between the leading vehicle and the following vehicles in the platoon. Each following CAV promptly transmits real-time information (speed and distance) to the leading vehicle. The leading vehicle computes and transmits optimal control decisions to each following vehicle to regulate their respective driving behaviors.

(4) We disregard delays related to actuation and information transmission, that is, the following CAVs can execute control decisions simultaneously.

### 2.2. CACC State-Space System Formulation

According to the assumptions, V2V communication exists between the leading vehicle 0 and the following vehicle *i* in Figure 2. Each Following CAV *i* transmits real-time state information gathered by its onboard sensors to the leading vehicle 0. The real-time state information gathered by the onboard sensors includes the relative distance $s_{i}$ between vehicle *i* and vehicle i−1, as well as the speed $v_{i}$ of vehicle *i*. The leading CAV 0 computes the optimal control decisions $u_{i}$ and transmits them to vehicle *i*, thereby regulating the driving behavior of the following vehicle *i*.

The core idea of cooperative-behavior-based CACC proposed in this paper is to achieve convergence among various state parameters [31]. The state parameters of the CAV platoon include the relative speed $\left(v_{i}\left(t\right)-v_{i-1}\left(t\right)\right)$ and the deviation-from-equilibrium distance $\left(s_{i}\left(t\right)-s_{t}^{*}\left(t\right)\right)$ [32]. Here, the deviation-from-equilibrium distance is defined as the disparity between the measured distance $s_{i}\left(t\right)$ and the ideal safe distance $s_{i}^{*}\left(t\right)$ for CAV *i* relative to CAV i−1. That is to say,

(1) $$\lim_{t\to \infty }\left(v_{i}\left(t\right)-v_{i-1}\left(t\right)\right)=0$$

(2) $$\lim_{t\to \infty }\left(s_{i}\left(t\right)-s_{t}^{*}\left(t\right)\right)=0$$

where $s_{i}\left(t\right)=d_{i-1}\left(t\right)-d_{i}\left(t\right)$. The $d_{i}\left(t\right)$ is the longitudinal position of vehicle *i* in the platoon at time *t*.

The ideal safe distance $s_{t}^{*}\left(t\right)$ is calculated as follows:

(3) $$s_{t}^{*}\left(t\right)=r_{i}^{*}\cdot v_{i}\left(t\right)+s_{f}$$

where $s_{f}$ is the safe distance of the CAV *i* to the predecessor vehicle. $r_{i}^{*}$ is the headway. The headway of each of the CAVs in the platoon stream is constant based on constant time headway (CTH) policy, that is, $r^{*}=r_{i}^{*}$.

We introduce the variables $\xi_{i}\left(t\right)$ and $\varsigma_{i}\left(t\right)$, which are

(4) $$\xi_{i}\left(t\right)=s_{i}\left(t\right)-s_{t}^{*}\left(t\right)$$

(5) $$\varsigma_{i}\left(t\right)=v_{i}\left(t\right)-v_{i-1}\left(t\right)$$

The Equation (4) can be rewritten as

(6) $$\xi_{i}\left(t\right)=d_{i-1}\left(t\right)-d_{i}\left(t\right)-r_{}^{*}\cdot v_{i}\left(t\right)-s_{f}$$

and then

(7) $${\dot{\xi }}_{i}\left(t\right)=-y_{i}\left(t\right)-r_{}^{*}\cdot a_{i}\left(t\right)$$

(8) $${\dot{\varsigma }}_{i}\left(t\right)=a_{i}\left(t\right)-a_{i-1}\left(t\right)$$

where $a_{i}\left(t\right)$ is the acceleration of vehicle *i* at time *t*

Denote $\xi \left(t\right)={\left[\xi_{1}\left(t\right),\xi_{2}\left(t\right),\cdots ,\xi_{n}\left(t\right)\right]}^{T}$, $\varsigma \left(t\right)={\left[\varsigma_{1}\left(t\right),\varsigma_{2}\left(t\right),\cdots ,\varsigma_{n}\left(t\right)\right]}^{T}$ and $u\left(t\right)={\left[a_{1}\left(t\right),a_{2}\left(t\right),\cdots ,a_{n}\left(t\right)\right]}^{T}$. ξ(t) and ς(t) are the state variables of the CAVs. u(t) is the optimal control decision. Then, the linear time-invariant system state-space model is defined as follows:

(9a) $$\begin{array}{cc} \; & \dot{X}\left(t\right)=\mathrm{AX}\left(t\right)+\mathrm{Bu}\left(t\right) \end{array}$$

(9b) $$\begin{array}{cc} \; & Y(t)=\mathrm{CX}(t)+V(t) \end{array}$$

where $X\left(t\right)={\left[\xi {\left(t\right)}^{T},\varsigma {\left(t\right)}^{T}\right]}^{T}$ are the state variables. $Y\left(t\right)={\left[\bar{\xi }{\left(t\right)}^{T},\bar{\varsigma }{\left(t\right)}^{T}\right]}^{T}$ are the observational variables of the system. $A=\left[\begin{array}{cc} 0_{n} & -I_{n}\\ 0_{n} & 0_{n} \end{array}\right]$, $B=\left[\begin{array}{c} M\\ S \end{array}\right]$, $C=\left[\begin{array}{cc} I_{n} & 0_{n}\\ 0_{n} & I_{n} \end{array}\right]$, $M=-r_{}^{*}\cdot I_{n}$, and $S=\left[\begin{array}{ccccc} 1 & 0 & 0 & \cdots & 0\\ -1 & 1 & 0 & \cdots & 0\\ 0 & -1 & 1 & \cdots & 0\\ 0 & 0 & \ddots & \ddots & 0\\ 0 & \cdots & 0 & -1 & 1 \end{array}\right]$. $I_{n}$ is *n*-dimensional identity matrix. 0 is the *n*-dimensional zero matrix.

Equation (9a) represents the state equation of the CAV platoon system, which describes the relationship between the deviation-from-equilibrium distance, the relative speed, and the acceleration of the CAVs in the platoon stream. Equation (9b) represents the observation equation, where Y(t) is the measurement of the onboard sensor at time t, and $V\left(t\right)\in {ℜ}^{2n}$ is themeasurement error.

### 2.3. Analysis of Onboard Sensor Measurement Errors

In this section, we discuss the impact mechanisms of the measurement errors. In real-world driving environments, the measurement values acquired by onboard sensors regarding the vehicle’s motion state are subject to measurement errors due to various factors such as outdoor temperature, rain, or foggy weather conditions; vehicle vibration; and sensor installation positions.

Let ${\bar{\xi }}_{i}\left(t\right)$ be the observed value representing the deviation-from-equilibrium distance:

(10) $$\begin{array}{cc} {\bar{\xi }}_{i}\left(t\right) & =\xi_{i}\left(t\right)+\varepsilon_{\xi_{i}}\left(t\right)\\ \; & ={\bar{s}}_{i}\left(t\right)-{\bar{s}}_{t}^{*}\left(t\right)\\ \; & =\left[s_{i}\left(t\right)+\varepsilon_{s_{i}}\left(t\right)\right]-\left[r^{*}\left(v_{i}\left(t\right)+\varepsilon_{v_{i}}\left(t\right)\right)+s_{f}\right]\\ \; & =\xi_{i}\left(t\right)+\left[\varepsilon_{s_{i}}\left(t\right)-r^{*}\varepsilon_{v_{i}}\left(t\right)\right] \end{array}$$

where $\varepsilon_{\xi_{i}}\left(t\right)$ is the measurement error of $\xi_{i}\left(t\right)$. ${\bar{s}}_{i}\left(t\right)$ is the measurement value of $s_{i}\left(t\right)$. $\varepsilon_{s_{i}}\left(t\right)$ is the measurement error of $s_{i}\left(t\right)$. ${\bar{s}}_{t}^{*}\left(t\right)$ is the measurement value of $s_{t}^{*}\left(t\right)$. $\varepsilon_{v_{i}}\left(t\right)$ is the measurement error of $v_{i}\left(t\right)$.

Similarly, let ${\bar{\varsigma }}_{i}\left(t\right)$ be the observed value representing the relative speed between vehicle *i* to its predecessor vehicle at time *t*:

(11) $$\begin{array}{cc} {\bar{\varsigma }}_{i}\left(t\right) & =\varsigma_{i}\left(t\right)+\varepsilon_{\varsigma_{i}}\left(t\right)\\ \; & ={\bar{v}}_{i}\left(t\right)-{\bar{v}}_{i-1}\left(t\right)\\ \; & =\left[v_{i}\left(t\right)+\varepsilon_{v_{i}}\left(t\right)\right]-\left[v_{i-1}\left(t\right)+\varepsilon_{v_{i-1}}\left(t\right)\right]\\ \; & =\varsigma_{i}\left(t\right)+\left[\varepsilon_{v_{i}}\left(t\right)-\varepsilon_{v_{i-1}}\left(t\right)\right] \end{array}$$

where $\varepsilon_{\varsigma_{i}}\left(t\right)$ is the observed error of $\varsigma_{i}\left(t\right)$. Then,

(12) $$\varepsilon_{\xi_{i}}\left(t\right)=\varepsilon_{s_{i}}\left(t\right)-r^{*}\varepsilon_{v_{i}}\left(t\right)$$

(13) $$\varepsilon_{\varsigma_{i}}\left(t\right)=\varepsilon_{v_{i}}\left(t\right)-\varepsilon_{v_{i-1}}\left(t\right)$$

According to the statistical principle, we assume that the measurement errors of $\varepsilon_{\xi_{i}}=\left[\varepsilon_{\xi_{i}}\left(1\right),\varepsilon_{\xi_{i}}\left(2\right),\cdots ,\varepsilon_{\xi_{i}}\left(m\right)\right]$ follow a normal distribution with a mean of zero and a variance of $\sigma_{\xi_{i}}^{2}$ with the increase in the number of measurements. Similarly, the measurement errors of $\varepsilon_{\varsigma_{i}}=\left[\varepsilon_{\varsigma_{i}}\left(1\right),\varepsilon_{\varsigma_{i}}\left(2\right),\cdots ,\varepsilon_{\varsigma_{i}}\left(m\right)\right]$ follow a normal distribution with a mean of zero and a variance of $\sigma_{v_{i}}^{2}$, i.e.,

(14) $$\varepsilon_{\xi_{i}}∼N\left(0,\sigma_{\xi_{i}}^{2}\right)$$

(15) $$\varepsilon_{\varsigma_{i}}∼N\left(0,\sigma_{\varsigma_{i}}^{2}\right)$$

According to th second assumption, $\varepsilon_{\xi_{i}}$ and $\varepsilon_{\varsigma_{i}}$ are independently and identically distributed. Therefore, let $\sigma_{\xi }^{2}=\sigma_{\xi_{i}}^{2}$, $\sigma_{\varsigma }^{2}=\sigma_{\varsigma_{i}}^{2}$, $i\in \left\{0,1,2,\cdots ,n\right\}$, where $\sigma_{\xi }$ and $\sigma_{\varsigma }$ are constants.

**Proposition** **1.** *The linear combination of independent normal random vectors with zero mean maintains normality.*

**Proof** **of Proposition 1**. Denote $X={\left[\varepsilon_{1},\varepsilon_{2},\cdots ,\varepsilon_{n}\right]}^{T}$; $\varepsilon_{i}$ represents the independent normal random vectors with means of zero. $\alpha =\left[\alpha_{1},\alpha_{2},\cdots ,\alpha_{n}\right]$, where $\alpha_{i}$ is a constant. Thus, the linear combination can be rewritten as

(16) $$\alpha X=\alpha_{1}\varepsilon_{1}+\alpha_{2}\varepsilon_{2}+\cdots +\alpha_{n}\varepsilon_{n}$$

The characteristic function is

(17) $$\begin{array}{cc} \varphi_{\alpha X}\left(t\right) & =\varphi_{\alpha_{1}\varepsilon_{1}}\left(t\right)\times \varphi_{\alpha_{2}\varepsilon_{2}}\left(t\right)\times \cdots \times \varphi_{\alpha_{n}\varepsilon_{n}}\left(t\right)\\ \; & =\exp\left(-\frac{\alpha_{1}^{2}\sigma_{1}^{2}t^{2}}{2}\right)\times \exp\left(-\frac{\alpha_{2}^{2}\sigma_{2}^{2}t^{2}}{2}\right)\times \cdots \times \exp\left(-\frac{\alpha_{n}^{2}\sigma_{n}^{2}t^{2}}{2}\right)\\ \; & =\exp\left(-\frac{\left(\alpha_{1}^{2}\sigma_{1}^{2}+\alpha_{2}^{2}\sigma_{2}^{2}+\cdots +\alpha_{n}^{2}\sigma_{n}^{2}\right)t^{2}}{2}\right) \end{array}$$

Thus, the linear combination of independent normal random vectors follows a normal distribution with a mean of zero and a variance of $\left(\alpha_{1}^{2}\sigma_{1}^{2}+\alpha_{2}^{2}\sigma_{2}^{2}+\cdots +\alpha_{n}^{2}\sigma_{n}^{2}\right)$. □

According to Proposition 1 and Equations (12) and (13), the Equations (14) and (15) can be written as

(18) $$\varepsilon_{\xi_{i}}\left(t\right)∼N\left(0,\sigma_{\xi }^{2}+r^{*2}\sigma_{\varsigma }^{2}\right)$$

(19) $$\varepsilon_{\varsigma_{i}}\left(t\right)∼N\left(0,2\sigma_{\varsigma }^{2}\right)$$

Therefore, V(t) follows a normal distribution with a mean of zero and a variance of R in the observation Equation (9b), and

(20) $$R=\left[\begin{array}{cc} \left(\sigma_{s}^{2}+r^{*2}\sigma_{v}^{2}\right)\cdot I_{n} & 0_{n}\\ 0_{n} & 2\sigma_{v}^{2}\cdot I_{n} \end{array}\right]$$

For CAV platoon systems, the measurement errors of the motion state produce a cumulative effect, that is, the variance in the CAVs platoon’s motion-state measurement errors is greater than the variance in the measurement errors from individual sensors. The cumulative effect has a significant negative impact on the CAVs’ platoon control, thereby necessitating the implementation of effective measures to mitigate the interference caused by measurement errors.

## 3. CAV Platoon Motion-State Estimation Based on Kalman Filtering

In this section, Kalman filtering is employed to estimate the motion state of the CAVs’ platoon systerm, thus aiming to mitigate the negative impact of the cumulative effect of the measurement errors. By applying the Euler formula, we derive the equivalent discrete state-space equation corresponding to the linear time-invariant system state-space model (9):

(21a) $$\begin{array}{cc} \; & X(t+\tau )=\Phi X(t)+\Gamma u(t) \end{array}$$

(21b) $$\begin{array}{cc} \; & Y(t)=\mathrm{CX}(t)+V(t) \end{array}$$

where $\Phi =I_{n}+\tau \cdot A$, Γ=τ·B, and τ is the sampling time.

The Kalman filtering state estimation consists of two steps. The first step is called a priori estimation:

(22) $$\hat{X}\left(\left.t+\tau \right|t\right)=\Phi \hat{X}\left(\left.t\right|t\right)+\Gamma u\left(t\right)$$

where $\hat{X}\left(\left.t+\tau \right|t\right)={\left[\hat{\xi }{\left(\left.t+\tau \right|t\right)}^{T},\hat{\varsigma }{\left(\left.t+\tau \right|t\right)}^{T}\right]}^{T}$ is the prior estimated state of CAV *i* for next time step t+τ, which is predicted based on the vehicle dynamics model Equation (9a) with the control input u(t) at the current time step *t*.

The second step is called a posteriori estimation:

(23) $$\hat{X}\left(\left.t+\tau \right|t+\tau \right)=\hat{X}\left(\left.t+\tau \right|t\right)+K\left(t+\tau \right)\left[Y\left(t+\tau \right)-C\hat{X}\left(\left.t+\tau \right|t\right)\right]$$

where $\hat{X}\left(\left.t+\tau \right|t+\tau \right)$ is the posterior estimated state vector. K(t+τ) is the Kalman gain.

Equation (23) employs the Kalman filter gain to correct the a priori estimate $\hat{X}\left(\left.t+\tau \right|t\right)$ by reducing the disparity between the actual measurement value Y(t+τ) and the estimated value $\hat{Y}\left(\left.t+\tau \right|t\right)$ of the CAVs’ platoon motion state at time t+τ. The estimated value is

(24) $$\hat{Y}\left(\left.t+\tau \right|t\right)=C\hat{X}\left(\left.t+\tau \right|t\right)$$

and the Kalman gain is

(25) $$K\left(t+\tau \right)=P\left(\left.t+\tau \right|t\right)C^{T}{\left[\mathrm{CP}\left(\left.t+\tau \right|t\right)C^{T}+R\right]}^{-1}$$

where

(26) $$P\left(\left.t+\tau \right|t\right)=\Phi P\left(\left.t\right|t\right)\Phi^{T}$$

(27) $$P\left(\left.t+\tau \right|t+\tau \right)=\left[I_{2n}-K\left(t+\tau \right)C\right]P\left(\left.t+\tau \right|t\right)$$

By substituting Equation (22) into Equation (23), we derive the Kalman filter discrete state estimator as follows:

(28) $$\hat{X}\left(\left.t+\tau \right|t+\tau \right)=\left(I_{n}+\tau A\right)\hat{X}\left(\left.t\right|t\right)+\Gamma u\left(t\right)+K\left(t+\tau \right)\left[Y\left(t+\tau \right)-C\left(\left(I_{n}+\tau A\right)\hat{X}\left(\left.t\right|t\right)+\Gamma u\left(t\right)\right)\right]$$

Equation (9a) is a continuous state-space equation. Therefore, it is necessary to convert the discrete Kalman state estimator into a continuous Kalman state estimator. Both sides of Equation (28) are subtracted by x and then divided by t, which can be written as

(29) $$\frac{\hat{X}\left(\left.t+\tau \right|t+\tau \right)-\hat{X}\left(\left.t\right|t\right)}{\tau }=A\hat{X}\left(\left.t\right|t\right)+\Gamma u\left(t\right)+\frac{K(t+\tau )}{\tau }\left[Y\left(t+\tau \right)-C\left(\left(I_{n}+\tau \cdot A\right)\hat{X}\left(\left.t\right|t\right)+\Gamma u\left(t\right)\right)\right]$$

Let $K\left(t\right)=\frac{K(t+\tau )}{\tau }$ and τ→0. Then, taking the limit on both sides of Equation (29) yields the optimal estimate of the linear continuous system for Equation (9a):

(30) $$\dot{\hat{X}}\left(t\right)=\left(A-K(t)C\right)\hat{X}\left(t\right)+\left(I_{2n}-K\left(t\right)C\right)\Gamma u\left(t\right)+K\left(t\right)Y\left(t\right)$$

## 4. Control Strategy and Solving Algorithms

### 4.1. Formulation of Optimal Control

This paper formulates the following optimal control problem to optimize the control decisions for all follower CAVs in the platoon at each sampling time τ, thereby improving the performance of CAVs platoon stream:

(31a) $$\begin{array}{c} \min_{u}\int_{0}^{T_{p}}\frac{1}{2}\left(\hat{X}{\left(t\right)}^{T}\left[\begin{array}{cc} R_{1} & \\ & R_{2} \end{array}\right]\hat{X}\left(t\right)+u{\left(t\right)}^{T}R_{3}u\left(t\right)\right)dt+\frac{1}{2}\hat{X}{\left(T_{p}\right)}^{T}\left[\begin{array}{cc} R_{4} & \\ & R_{5} \end{array}\right]\hat{X}\left(T_{p}\right) \end{array}$$

(31b) $$\begin{array}{c} \dot{\hat{X}}\left(t\right)=\left(A-K(t)C\right)\hat{X}\left(t\right)+\left(I_{2n}-K\left(t\right)C\right)\Gamma u\left(t\right)+K\left(t\right)Y\left(t\right)\; \end{array}$$

(31c) $$\begin{array}{c} s_{i}\left(t\right)=x_{i}\left(t\right)+r^{*}\left(v_{0}\left(0\right)+\sum_{j=1}^{i}y_{j}\left(t\right)\right)+s_{f}\geq s_{\min}>0\; \end{array}$$

(31d) $$\begin{array}{c} 0\leq v_{i}\left(t\right)\leq v_{\max}\; \end{array}$$

(31e) $$\begin{array}{c} u_{\min}\leq u_{i}\left(t\right)\leq u_{\max}\; \end{array}$$

(31f) $$\begin{array}{c} X\left(0\right)={\left[{\xi_{0}}^{T},{\varsigma_{0}}^{T}\right]}^{T}\; \end{array}$$

(31g) $$\begin{array}{c} \forall i=1,2,\cdots ,n\; \end{array}$$

Equation (31a) is the the objective function, which is used to find the optimal control input $u^{*}\left(t\right)$ for the CAVs’ platoon system (9). The objective function consists of two components. In the first part, $\hat{X}{\left(t\right)}^{T}\left[\begin{array}{cc} R_{1} & \\ & R_{2} \end{array}\right]\hat{X}\left(t\right)$ is used to minimize the deviation-from-equilibrium distance and the relative speed between CAV *i* to CAV i−1 in the platoon. $u{\left(t\right)}^{T}R_{3}u\left(t\right)$ is incorporated with the intention of enhancing the comfort of the platoon operations by reducing emergency braking and sudden acceleration. The succeeding component, denoted as $\hat{X}{\left(T_{p}\right)}^{T}\left[\begin{array}{cc} R_{4} & \\ & R_{5} \end{array}\right]\hat{X}\left(T_{p}\right)$, assumes the role of the terminal cost function. This particular term serves the purpose of imposing penalization upon deviations of the state-variable values from the equilibrium point, thus effectively influencing the objective function. The weight matrices $R_{1}$, $R_{2}$, $R_{4}$, and $R_{5}$ are symmetric positive definite matrices. $R_{3}$ is a positive definite diagonal matrix. Equation (31b), which delineates the dynamic behavior of platoon systems over time, is the system-dynamics equation governing the state variables encompassing the equilibrium-distance discrepancies and the relative speed pertaining to every contiguous pair of CAVs in the platoon. Equation (31c) embodies the safety constraint, which serves the essential function of guaranteeing that the intervehicle spacing among any two successive vehicles within the platoon consistently exceeds the defined threshold, which is denoted as $s_{\min}$. Equation (31d) represents the speed constraint, where $v_{\max}$ is the maximum speed limit on the highway. Equation (31e) sets the upper and lower limits for acceleration. Equation (31f) represents the initial values of the state variables.

### 4.2. Solution Algorithm

In this section, a two-point boundary-value problem has been formulated to address the optimal control problem as stated in (31); its resolution dictates the optimal control decisions for the CAVs platoon. The optimal control problem (31) is characterized by the inclusion of pure-state variable inequality constraints (31c). The incorporation of these pure-state variable inequality constraints notably compounds the intricacy for devising an efficient solution algorithm, primarily due to their dependency on the historical sequence of the control actions. In order to tackle this challenge, the initial optimal control problem (31) has been transformed into an equivalent problem that circumvents the presence of pure-state variable inequality constraints. To achieve this, a new variable $X_{N}$ is introduced, thus establishing a functional relationship as follows.

(32a) $$\begin{array}{c} {\dot{X}}_{N}\left(t\right)=\sum_{i=1}^{n}\left(X_{N,1}^{i}\left(t\right),X_{N,2}^{i}\left(t\right),X_{N,3}^{i}\left(t\right)\right)\; \end{array}$$

(32b) $$\begin{array}{c} X_{N,1}^{i}\left(t\right)={\left(s_{i}\left(t\right)-s_{\min}\right)}^{2}\delta \left(s_{i}\left(t\right)-s_{\min}\right),\delta \left(s_{i}\left(t\right)-s_{\min}\right)=\left\{\begin{array}{cc} 0 & ifs_{i}\left(t\right)-s_{\min}\geq 0\\ 1 & otherwise \end{array}\right.\; \end{array}$$

(32c) $$\begin{array}{c} X_{N,2}^{i}\left(t\right)={\left(v_{\max}-v_{i}\left(t\right)\right)}^{2}\delta \left(v_{\max}-v_{i}\left(t\right)\right),\delta \left(v_{\max}-v_{i}\left(t\right)\right)=\left\{\begin{array}{cc} 0 & ifv_{\max}-v_{i}\left(t\right)\geq 0\\ 1 & otherwise \end{array}\right. \end{array}$$

(32d) $$\begin{array}{c} X_{N,3}^{i}\left(t\right)=v_{i}\left(t\right)\delta \left(v_{i}\left(t\right)\right),\delta \left(v_{i}\left(t\right)\right)=\left\{\begin{array}{cc} 0 & ifv_{i}\left(t\right)\geq 0\\ 1 & otherwise \end{array}\right.\; \end{array}$$

The optimal control problem (31) can be reformulated as follows:

(33a) $$\begin{array}{c} \min_{u}\int_{0}^{T_{p}}\frac{1}{2}\left(\hat{X}{\left(t\right)}^{T}\left[\begin{array}{cc} R_{1} & \\ & R_{2} \end{array}\right]\hat{X}\left(t\right)+u{\left(t\right)}^{T}R_{3}u\left(t\right)\right)dt+\frac{1}{2}\hat{X}{\left(T_{p}\right)}^{T}\left[\begin{array}{cc} R_{4} & \\ & R_{5} \end{array}\right]\hat{X}\left(T_{p}\right)+\gamma {\left(\hat{X}\left(T_{p}\right)\right)}^{2} \end{array}$$

(33b) $$\begin{array}{c} \dot{\hat{X}}\left(t\right)=\left(A-K(t)C\right)\hat{X}\left(t\right)+\left(I_{2n}-K\left(t\right)C\right)\Gamma u\left(t\right)+K\left(t\right)Y\left(t\right)\; \end{array}$$

(33c) $$\begin{array}{c} {\dot{X}}_{N}\left(t\right)=\sum_{i=1}^{n}\left(X_{N,1}^{i}\left(t\right),X_{N,2}^{i}\left(t\right),X_{N,3}^{i}\left(t\right)\right)\; \end{array}$$

(33d) $$\begin{array}{c} u_{\min}\leq u_{i}\left(t\right)\leq u_{\max}\; \end{array}$$

(33e) $$\begin{array}{c} X\left(0\right)={\left[{\xi_{0}}^{T},{\varsigma_{0}}^{T}\right]}^{T}\; \end{array}$$

(33f) $$\begin{array}{c} X_{N}\left(0\right)=0\; \end{array}$$

The Hamiltonian equation for the optimal control problem (33) is

(34) $$\begin{array}{c} H\left(\hat{X}\left(t\right),\lambda_{A}\left(t\right),u\left(t\right)\right)=\hat{X}{\left(t\right)}^{T}\left[\begin{array}{cc} R_{1} & \\ & R_{2} \end{array}\right]\hat{X}\left(t\right)+u{\left(t\right)}^{T}R_{3}u\left(t\right)+\lambda {\left(t\right)}^{T}\left(A\hat{X}\left(t\right)+\mathrm{Bu}\left(t\right)\right)\\ +\lambda_{N}\left(t\right)\left(\sum_{i=1}^{n}\left(X_{N,1}^{i}\left(t\right),X_{N,2}^{i}\left(t\right),X_{N,3}^{i}\left(t\right)\right)\right) \end{array}$$

where $\lambda \left(t\right)={\left[\lambda_{1}\left(t\right),\lambda_{2}\left(t\right),\cdots ,\lambda_{2n}\left(t\right)\right]}^{T}$ and $\lambda_{N}\left(t\right)$ are the costate variables. Then, let $\lambda_{A}\left(t\right)={\left[\lambda {\left(t\right)}^{T},\lambda_{N}\left(t\right)\right]}^{T}$, and let $X_{A}\left(t\right)={\left[\hat{X}{\left(t\right)}^{T},X_{N}\left(t\right)\right]}^{T}$. In accordance with Pontryagin’s minimum principle, the requisite conditions for $u^{*}\left(t\right)$ to constitute an optimal solution for the problem (34) are defined as follows:

(35) $${\dot{\lambda }}_{A}\left(t\right)=-\frac{\partial H}{\partial X_{A}\left(t\right)}$$

with the initial conditions given in Equation (33e) and the terminal conditions as

(36) λ(Tp)=∂12X^(t)TR4R5X^(t)/∂12X^(t)TR4R5X^(t)∂X^(t)∂X^(t)t=Tp=R4R5X^(Tp)

(37) λN(Tp)=∂γXN(t)2/∂γXN(t)2∂∂XN(t)t=Tp=2γXN(Tp)

Finally, the following initial and terminal conditions are derived, thus forming a two-point boundary-value problem:

(38a) $$\begin{array}{c} \dot{\hat{X}}\left(t\right)=\left(A-K(t)C\right)\hat{X}\left(t\right)+\left(I_{2n}-K\left(t\right)C\right)\Gamma u\left(t\right)+K\left(t\right)Y\left(t\right) \end{array}$$

(38b) $$\begin{array}{c} {\dot{X}}_{N}\left(t\right)=\sum_{i=1}^{n}\left(X_{N,1}^{i}\left(t\right),X_{N,2}^{i}\left(t\right),X_{N,3}^{i}\left(t\right)\right)\; \end{array}$$

(38c) $$\begin{array}{c} \dot{\lambda }\left(t\right)=-\left[\begin{array}{cc} R_{1} & \\ & R_{2} \end{array}\right]\hat{X}\left(t\right)-\lambda {\left(t\right)}^{T}A-\left[\begin{array}{c} c_{x}\\ c_{y} \end{array}\right]\lambda_{N}\left(t\right)\; \end{array}$$

(38d) $$\begin{array}{c} {\dot{\lambda }}_{N}\left(t\right)=0\; \end{array}$$

(38e) $$\begin{array}{c} X\left(0\right)={\left[{\xi_{0}}^{T},{\varsigma_{0}}^{T}\right]}^{T}\; \end{array}$$

(38f) $$\begin{array}{c} X_{N}\left(0\right)=0\; \end{array}$$

(38g) $$\begin{array}{c} \lambda \left(T_{p}\right)=\left[\begin{array}{cc} R_{4} & \\ & R_{5} \end{array}\right]\hat{X}\left(T_{p}\right)\; \end{array}$$

(38h) $$\begin{array}{c} \lambda_{N}\left(T_{p}\right)=2\gamma X_{N}\left(T_{p}\right)\; \end{array}$$

where

$$c_{x}=\frac{\partial {\dot{X}}_{N}\left(t\right)}{\partial \hat{X}\left(t\right)}=2\left[\begin{array}{c} \left(s_{1}\left(t\right)-s_{\min}\right)\delta \left(s_{1}\left(t\right)-s_{\min}\right)\\ \left(s_{2}\left(t\right)-s_{\min}\right)\delta \left(s_{2}\left(t\right)-s_{\min}\right)\\ \vdots \\ \left(s_{n}\left(t\right)-s_{\min}\right)\delta \left(s_{n}\left(t\right)-s_{\min}\right) \end{array}\right]$$

$$c_{y}=\frac{\partial {\dot{X}}_{N}\left(t\right)}{\partial y(t)}=2\left[\begin{array}{c} \left(v_{\max}-v_{1}\left(t\right)\right)\delta \left(v_{\max}-v_{1}\left(t\right)\right)\\ \left(v_{\max}-v_{2}\left(t\right)\right)\delta \left(v_{\max}-v_{2}\left(t\right)\right)\\ \vdots \\ \left(v_{\max}-v_{n}\left(t\right)\right)\delta \left(v_{\max}-v_{n}\left(t\right)\right) \end{array}\right]+2\left[\begin{array}{c} v_{1}\left(t\right)\delta \left(v_{1}\left(t\right)\right)\\ v_{2}\left(t\right)\delta \left(v_{2}\left(t\right)\right)\\ \vdots \\ v_{n}\left(t\right)\delta \left(v_{n}\left(t\right)\right) \end{array}\right]+\left[\begin{array}{c} c_{1,y}\\ c_{2,y}\\ \vdots \\ c_{n,y} \end{array}\right]$$

$$c_{i,y}=\sum_{j=1}^{i}2\left(s_{j}\left(t\right)-s_{\min}\right)\delta \left(s_{j}\left(t\right)-s_{\min}\right),\forall i=1,2,\cdots ,n$$

The CAV platoon cooperative behavioral control problem (31) is transformed into a two-point boundary-value problem (38), which can be solved using various methods [33]. In this paper, the shooting method [34] is employed to solve the two-point boundary-value problem (38), thus obtaining the unique solution for the following optimal control decisions:

(39) $$u_{i}^{*}\left(t\right)=\left\{\begin{array}{c} u_{\min},\\ u_{\max},\\ p_{i}\left(t\right), \end{array}\begin{array}{c} if\\ if\\ if \end{array}\right.\begin{array}{c} p_{i}\left(t\right)<u_{\min}\\ p_{i}\left(t\right)>u_{\max}\\ u_{\min}\leq p_{i}\left(t\right)\leq u_{\max} \end{array}$$

where $P={\left[p_{1}\left(t\right),p_{2}\left(t\right),\cdots ,p_{n}\left(t\right)\right]}^{T}=-R_{3}^{-1}\left(B^{T}\lambda^{*}\left(t\right)\right)$.

## 5. Simulation Experiment and Analysis

### 5.1. Experiment Setup

**Figure 3:**
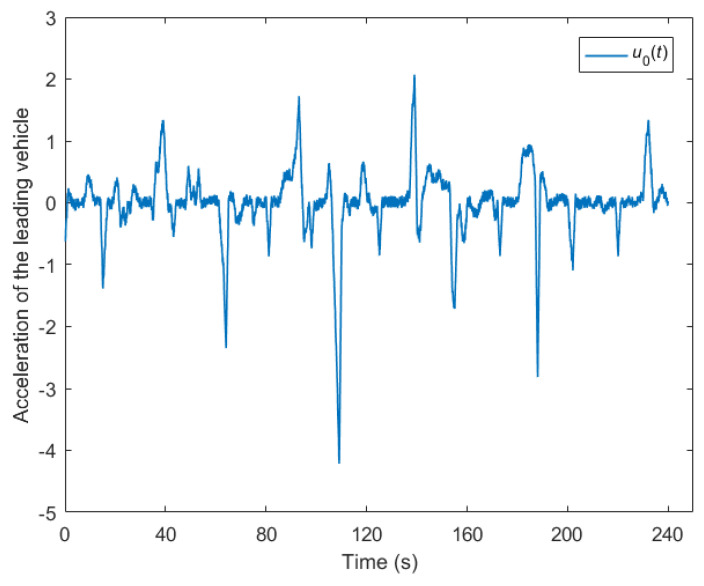
Acceleration of the leading vehicle.

This section conducts numerical experiments to demonstrate the efficacy of the proposed cooperative control method of the CAVs’ platoon system. The platoon under consideration comprises eight CAVs, wherein one serves as the leading CAV, denoted as (i=0), while the remaining seven function as following CAVs. The movement of the leading CAV in the experiment has been extrapolated from NGSIM field data [35], as depicted in Figure 3. This dataset encompasses a four-minute record with a resolution 0.1 s of the vehicle trajectories gathered along the eastbound I-80 route in Emeryville, San Francisco, California. A consistent time headway of 1 s was employed across the entire platoon in order to mitigate any disparities in the controller transient response, thus facilitating a uniform traffic flow. The initial conditions for the numerical experiments were set as follows: the initial acceleration $a_{i}\left(0\right)$ was 0 m/s${}^{2}$, and the initial speed $v_{i}\left(0\right)$ was 25 m/s for all the CAVs in the platoon; the safe distance $s_{f}$ of CAV i to the predecessor CAV i-1 was set to 10 m; The sampling time was set to τ=0.1 s; according to the numerical experiment in Ploeg et al., the measurement noise of the sensors were set to $\sigma_{s}=0.17$ and $\sigma_{v}=0.13$ [36]; the vehicle acceleration range was set to [−5 m/s${}^{2}$, 3 m/s${}^{2}$]; the maximum speed limit $v_{\max}$ on the highway was set to 120 km/h.

### 5.2. Experimental Results and Analysis

#### 5.2.1. Control Decisions and Performance of Each CAV in the Platoon

**Figure 4:**
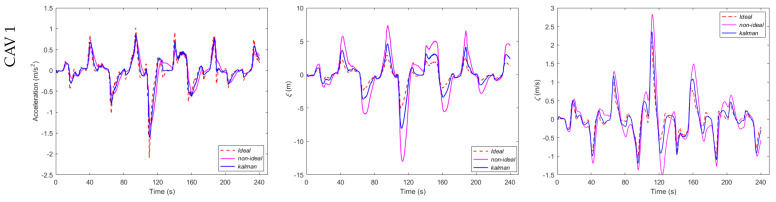

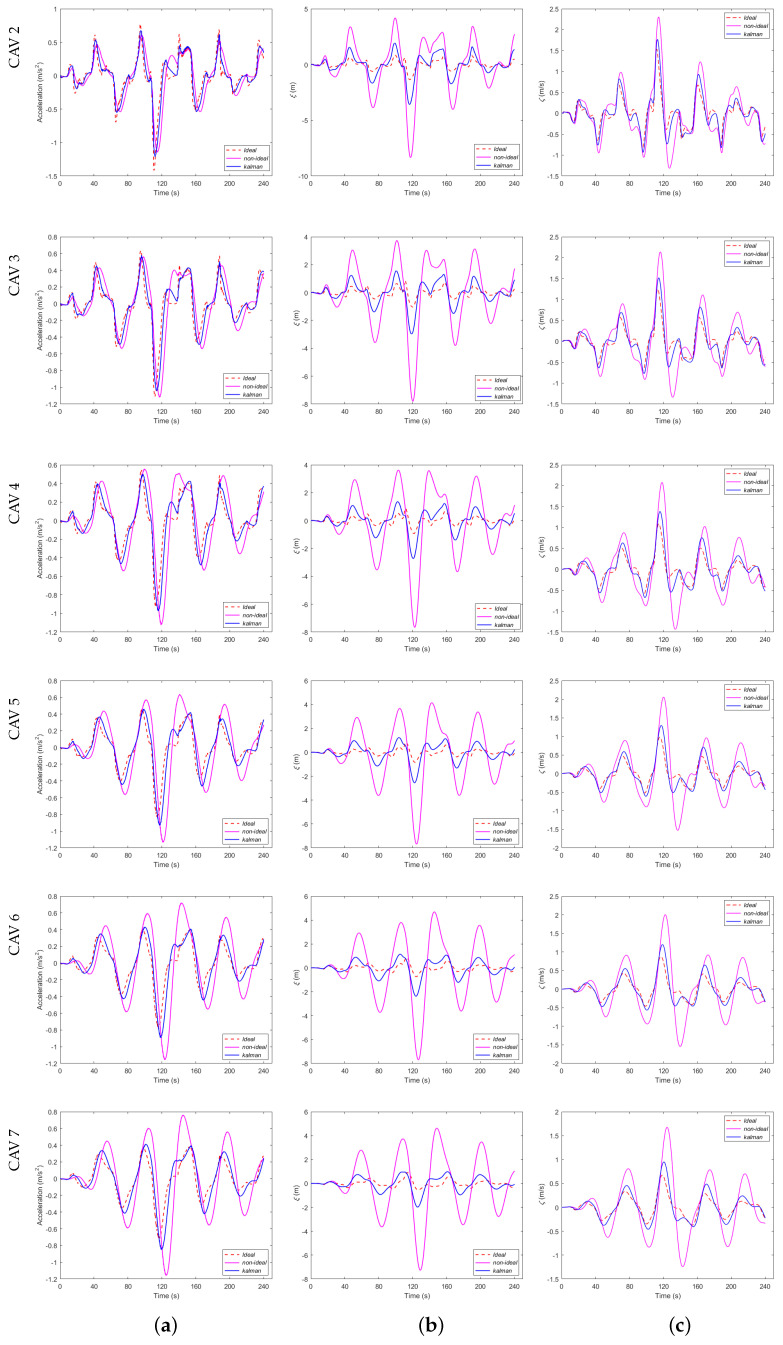
Control decisions and performance of a single CAV in the platoon: (**a**) control decisions; (**b**) deviation-from-equilibrium distance; (**c**) relative speed.

This section is dedicated to the evaluation of the control decisions and the performance of an individual CAV within the platoon stream. Figure 4 serves as an illustrative representation of the control decisions and performance of a single CAV operating within the platoon. To facilitate a meaningful comparison between different scenarios, Figure 4 incorporates three distinct curves, each of which represents a unique case. In Case 1, we considered an ideal condition where the platoon state data remained unaffected by any measurement errors. The red dotted line in Figure 4 corresponds to this scenario. In Case 2, we introduced the influence of measurement errors originating from the onboard sensor, thereby reflecting a more realistic scenario. The pink solid line in Figure 4 illustrates the control decisions and performance under the impact of sensor measurement errors. Case 3 represents an approach to mitigate the impact of sensor measurement errors. Here, we applied Kalman filtering to the motion state data within the platoon formation. The filtered data significantly reduced the adverse effects of the sensor measurement errors. The blue solid line in Figure 4 corresponds to this scenario.

Figure 4a visually represents the control decisions for the three distinct cases, spanning from CAV 1 to CAV 7 within the platoon. It is evident that the control decisions for the CAV 1 remained relatively consistent across all three conditions. However, as we observed the accumulation of measurement errors, the disparity in the control decisions between Case 1 and Case 3 remained small, while the oscillation range of the control decisions in Case 3 noticeably expanded. This observation underscores the adverse impact of cumulative error effects on the control of trailing CAVs within the platoon stream. Notably, a significant acceleration error tended to introduce instability into the platoon’s control. Figure 4b offers a comparative analysis of the deviation from the equilibrium distance for the three different cases. It is discernible that the deviation from the equilibrium distance between Case 2 and Case 1 was more pronounced than the deviation observed between Case 3 and Case 1. This disparity emphasizes that the presence of measurement errors, as depicted in Case 2, leads to a greater deviation from the desired equilibrium distance, thereby adversely affecting platoon stability. Furthermore, Figure 4c illustrates the relative speed between the three cases. Here, we again note that the relative speed difference between Case 2 and Case 1 was more significant than that observed between Case 3 and Case 1. This finding further accentuates the influence of measurement errors, particularly in Case 2, where a higher relative speed deviation posed challenges to the platoon control. In summary, Figure 4 provides valuable insights into the impact of the measurement errors on the control decisions, equilibrium distance, and relative speed within the CAV platoon. These findings underscore the importance of our proposed algorithm in mitigating the adverse effects of sensor measurement errors and enhancing the stability of CAV platoons.

#### 5.2.2. Control Decisions and Performance of the CAV Platoon Stream

**Figure 5:**
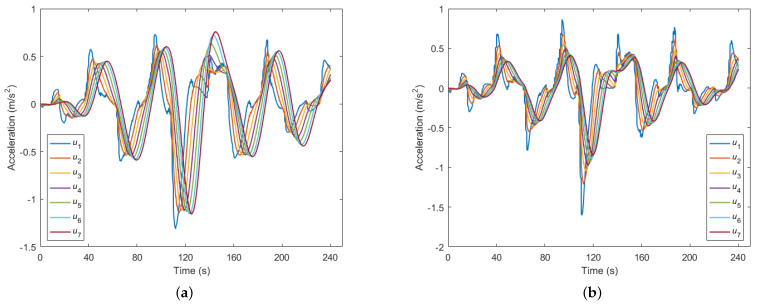
Optimal control decisions of the following CAVs: (**a**) affected by sensor measurement errors; (**b**) eliminating sensor measurement errors with Kalman filtering.

This section is dedicated to a comprehensive comparison of the control decisions and control performance outcomes within the CAV platoon stream. Figure 5 showcases the optimal control decisions for the following vehicles in the CAV platoon, which were estimated using the proposed method. As shown in Figure 5a, we delved into the estimated control decisions influenced by measurement errors. Notably, the control decisions for the CAV 1 exhibited a marginally reduced oscillation amplitude when compared to the leading vehicle. However, as we progressed through the subsequent trailing CAVs, it became evident that they manifested slightly larger oscillation amplitudes in their optimal control decisions relative to their preceding vehicles. Transitioning to Figure 5b, we observed that the estimated control decisions underwent Kalman filtering to mitigate the impact of sensor measurement inaccuracies. Here, the amplitudes of the control decisions for the subsequent CAVs exhibited a progressive reduction relative to that of the CAV 1. This signified a gradual attenuation of the traffic oscillations as we moved from the lead to the rear of the platoon stream. Furthermore, the effectiveness of our cooperative control approach became particularly evident when examining instances of sudden acceleration or deceleration maneuvers, as observed at 70 s, 110 s, 140 s, and 190 s. During these intervals, a noticeable reduction in the acceleration magnitude was observed in the subsequent vehicles. This observation underscores the capability of our proposed approach to significantly enhance ride comfort within the CAV platoon.

**Figure 6:**
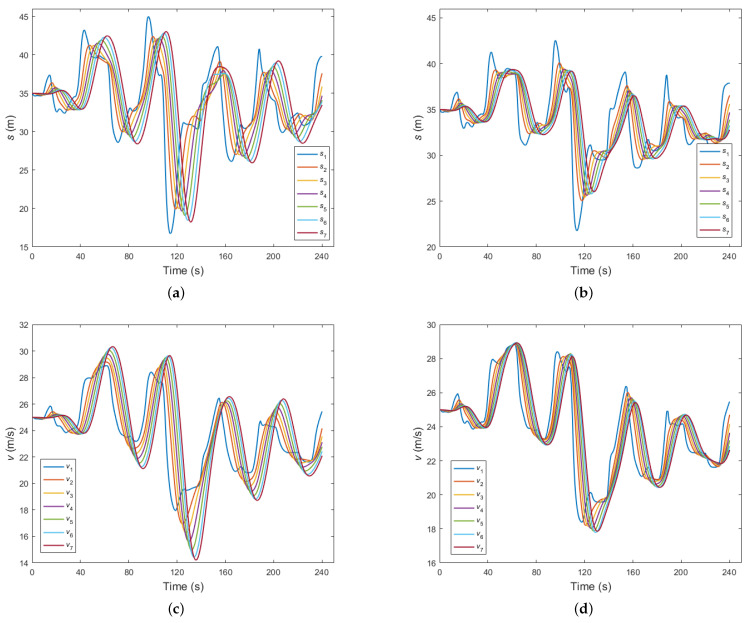
Optimal equilibrium spacing and speed of the following CAVs: (**a**) equilibrium distance affected by sensor measurement errors; (**b**) equilibrium distance unaffected by measurement errors; (**c**) speed affected by sensor measurement errors; (**d**) speed unaffected by measurement errors.

Figure 6 serves as an illustrative representation of the optimal equilibrium spacing and speed of the trailing vehicles within the CAV platoon. As shown in Figure 6a,c, we scrutinized the scenarios where the Kalman filter was not employed to eliminate measurement errors. Here, we observed a sequential increase in the oscillations of the equilibrium spacing and speed among the following vehicles within the platoon. This notable trend underscores the adverse impact of measurement errors on the control strategy, thus resulting in suboptimal control performance within the CAV platoon system. Conversely, when we introduced the application of the Kalman filter to mitigate measurement inaccuracies, as demonstrated in Figure 6b,d, the outcome was strikingly different. The effectiveness of the Kalman filter became apparent, as it efficiently suppressed oscillations in both the optimal equilibrium distances and speeds among the following vehicles in the platoon. This outcome highlights the pivotal role played by the Kalman filter in enhancing the robustness and stability of the control strategy, thereby ultimately leading to improved control performance within the CAV platoon.

**Figure 7:**
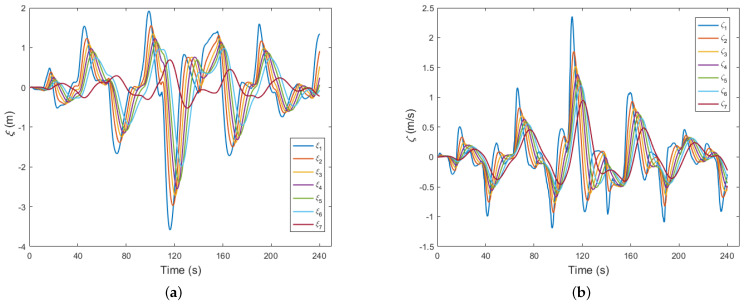
Control performance of CAV platoon: (**a**) deviation-from-equilibrium distance; (**b**) relative speed.

Figure 7 offers a detailed representation of the control performance associated with deviations from the equilibrium spacing and relative speed, which is a crucial aspect governed by the cooperative control strategy implemented within the CAV platoon system introduced in this study. As shown in Figure 7a, we scrutinized the deviation-from-equilibrium distance for the trailing CAVs. Notably, the amplitude of oscillation in the deviation-from-equilibrium distance gradually diminished as one progressed from the leading vehicle towards the rear of the platoon. This gradual reduction culminated in the oscillation curve of the seventh following vehicle converging towards a value of zero. As shown in Figure 7b, we delved into the relative speed between each following vehicle and its preceding counterpart. Here again, we observed a consistent trend of diminishing oscillation amplitudes as we traversed from the leading end to the trailing end of the platoon. This trend suggests a convergence of the motion state within the CAV platoon towards a state of equilibrium. Of noteworthy significance is the robustness of this convergence, even in scenarios where real-world disturbances were introduced, such as the relatively extreme driving maneuvers undertaken by the lead vehicle at 70 s, 110 s, and 140 s. Despite these challenging events causing observable oscillations in the motion state of the CAV platoon, they swiftly stabilized and returned to equilibrium conditions. The simulation results in Figure 7 also validate the core proposition put forth in this paper, as expressed by Equations (1) and (2), wherein the equilibrium distance and the ideal safe distance ultimately converge. In conclusion, the methodology presented in this paper enables the effective coordination of following vehicles within the CAV platoon system, thereby facilitating seamless deceleration and acceleration actions. By doing so, it efficiently mitigates oscillations in the operation of the convoy, thereby ensuring the overall stability and reliability of the CAV platoon.

## 6. Conclusions

This paper has demonstrated the cumulative impact of measurement errors in vehicular sensors. It has established a mathematical model for CAV platoon operation that takes into account the measurement errors. Building upon this foundation, it has utilized Kalman filtering to mitigate the adverse effects of measurement errors and proposed an optimal control decision method for CAV platoons. Furthermore, it has transformed the problem into a two-point boundary-value problem to obtain a unique solution. Simulation results demonstrate that the cooperative control method proposed in this paper effectively coordinated the behavior of following vehicles within the platoon. It enabled smooth acceleration and deceleration, eliminated traffic oscillations in the convoy, and ensured the stability and comfort of the CAV platoon.

The study has primarily addressed the perspective of measurement errors in vehicular sensors. In future investigations, it will be imperative to adopt a more comprehensive approach that accounts for the influence of factors such as vehicular communication latency, vehicle actuator delay, computational latency, and others on cooperative control. This holistic consideration will lead to the development of a more realistic model for CACC in CAV platoons, thereby ultimately enhancing platoon stability in complex road environments. Furthermore, subsequent research endeavors should focus on the computational efficiency of cooperative control algorithms, with the aim of proposing more efficient algorithms to facilitate the practical implementation of CAV platoons.

## Data Availability

Not applicable.

## References

[1] Li K., Chang X., Li J., Xu Q., Gao B., Pan J. (2020). Cloud control system for intelligent and connected vehicles and its application. Automot. Eng..

[2] Zhou A., Peeta S., Wang J. (2023). Cooperative control of a platoon of connected autonomous vehicles and unconnected human-driven vehicles. Comput. Civ. Infrastruct. Eng..

[3] Wang Z., Bian Y., Shladover S.E., Wu G., Li S.E., Barth M.J. (2019). A survey on cooperative longitudinal motion control of multiple connected and automated vehicles. IEEE Intell. Transp. Syst. Mag..

[4] Dhawankar P., Agrawal P., Abderezzak B., Kaiwartya O., Busawon K., Raboacă M.S. (2021). Design and Numerical Implementation of V2X Control Architecture for Autonomous Driving Vehicles. Mathematics.

[5] Lazar R.-G., Pauca O., Maxim A., Caruntu C.-F. (2023). Control Architecture for Connected Vehicle Platoons: From Sensor Data to Controller Design Using Vehicle-to-Everything Communication. Sensors.

[6] Desjardins C., Chaib-Draa B. (2011). Cooperative adaptive cruise control: A reinforcement learning approach. IEEE Trans. Intell. Transp. Syst..

[7] Gong S., Shen J., Du L. (2016). Constrained optimization and distributed computation based car following control of a connected and autonomous vehicle platoon. Transp. Res. Part B Methodol..

[8] Zheng Y., Li S.E., Wang J., Cao D., Li K. (2015). Stability and scalability of homogeneous vehicular platoon: Study on the influence of information flow topologies. IEEE Trans. Intell. Transp. Syst..

[9] Liu C., Zhuang W.C., Yin G., Huang Z., Liu H. (2020). Cooperative merging control of multiple connected and automated vehicles on freeway ramp. J. Southeast Univ. (Natural Sci. Ed.).

[10] Chang X., Xu Q., Li K., Bi Y., Ha H., Zhang J. (2019). Analysis of intelligent and connected vehicle control under communication delay and packet loss. China J. Highw. Transp..

[11] Wang J., Lu L., Peeta S. (2022). Real-time deployable and robust cooperative control strategy for a platoon of connected and autonomous vehicles by factoring uncertain vehicle dynamics. Transp. Res. Part B Methodol..

[12] Ren P., Jiang H., Xu X. (2023). Research on a Cooperative Adaptive Cruise Control (CACC) Algorithm Based on Frenet Frame with Lateral and Longitudinal Directions. Sensors.

[13] Tan Y., Zhang K. (2022). Real-Time Distributed Cooperative Adaptive Cruise Control Model Considering Time Delays and Actuator Lag. Transp. Res. Rec..

[14] Wu J., Qu X. (2022). Intersection control with connected and automated vehicles: A review. J. Intell. Connect. Veh..

[15] Cui L., Hu J., Park B.B., Bujanovic P. (2018). Development of a simulation platform for safety impact analysis considering vehicle dynamics, sensor errors, and communication latencies: Assessing cooperative adaptive cruise control under cyber attack. Transp. Res. Part C Emerg. Technol..

[16] Tian M.-W., Yan S.-R., Mohammadzadeh A., Tavoosi J., Mobayen S., Safdar R., Assawinchaichote W., Vu M.T., Zhilenkov A. (2021). Stability of Interval Type-3 Fuzzy Controllers for Autonomous Vehicles. Mathematics.

[17] Ghaedi M., Bayat F., Fekih A., Mobayen S. (2020). Robust performance improvement of lateral motion in four-wheel independent-drive electric vehicles. IEEE Access.

[18] Li K., Li J., Chang X., Gao B., Xu Q., Li S. (2020). Principles and typical applications of cloud control system for intelligent and connected vehicles. J. Automot. Saf. Energy.

[19] Li Y., Hao G. (2023). Energy-Optimal Adaptive Control Based on Model Predictive Control. Sensors.

[20] Andrade E., Matos F., Santos A. (2023). A virtual models-based CAVs platoon resilient to network and sensor attacks. Ad Hoc Networks.

[21] Eskandarian A., Wu C., Sun C. (2019). Research advances and challenges of autonomous and connected ground vehicles. IEEE Trans. Intell. Transp. Syst..

[22] Wang B., Luo Y., Zhong Z., Li K. (2022). Robust non-fragile fault tolerant control for ensuring the safety of the intended functionality of cooperative adaptive cruise control. IEEE Trans. Intell. Transp. Syst..

[23] Cai B., Zhao L., Wang Q., Yan M., Fang T. (2023). A strategy of vehicle following on slope road at night considering the safety of the intended functionality. Phys. A Stat. Mech. Its Appl..

[24] Guo G., Yue W. (2014). Sampled-data cooperative adaptive cruise control of vehicles with sensor failures. IEEE Trans. Intell. Transp. Syst..

[25] Chen Z., Park B.B. (2019). Preceding vehicle identification for cooperative adaptive cruise control platoon forming. IEEE Trans. Intell. Transp. Syst..

[26] Yang T., Murguia C., Nešić D., Lv C. (2023). A Robust CACC Scheme Against Cyberattacks Via Multiple Vehicle-to-Vehicle Networks. IEEE Trans. Veh. Technol..

[27] Zhou Z., Li L., Qu X., Ran B. (2023). An autonomous platoon formation strategy to optimize CAV car-following stability under periodic disturbance. Phys. A Stat. Mech. Appl..

[28] Sheikh M., Peng Y. (2023). A Collision Avoidance Model for On-Ramp Merging of Autonomous Vehicles. KSCE J. Civ. Eng..

[29] Kuutti S., Bowden R., Jin Y., Barber P., Fallah S. (2020). A survey of deep learning applications to autonomous vehicle control. IEEE Trans. Intell. Transp. Syst..

[30] Selvaraj D.C., Hegde S., Amati N., Deflorio F., Chiasserini C.F. (2023). A Deep Reinforcement Learning Approach for Efficient, Safe and Comfortable Driving. Appl. Sci..

[31] Swaroop D., Hedrick J.K. (1999). Constant spacing strategies for platooning in automated highway systems. J. Dyn. Syst. Meas. Control..

[32] Wang M., Daamen W., Hoogendoorn S.P., van Arem B. (2014). Rolling horizon control framework for driver assistance systems. Part I: Mathematical formulation and non-cooperative systems. Transp. Res. Part C Emerg. Technol..

[33] Keller H.B. (1976). Numerical Solution of Two Point Boundary Value Problems.

[34] Wang J., Gong S., Peeta S., Lu L. (2019). A real-time deployable model predictive control-based cooperative platooning approach for connected and autonomous vehicles. Transp. Res. Part B Methodol..

[35] Next Generation Simulation (2007). U.S. Department of Transportation. https://ops.fhwa.dot.gov/trafficanalysistools/ngsim.htm.

[36] Zhou A., Gong S., Wang C., Peeta S. (2020). Smooth-switching control-based cooperative adaptive cruise control by considering dynamic information flow topology. Transp. Res. Rec..

